# Phase II trial of carboplatin and bevacizumab in patients with breast cancer brain metastases

**DOI:** 10.1186/s13058-020-01372-w

**Published:** 2020-11-30

**Authors:** Jose Pablo Leone, Kyrre E. Emblem, Michelle Weitz, Rebecca S. Gelman, Bryan P. Schneider, Rachel A. Freedman, Jerry Younger, Marco C. Pinho, A. Gregory Sorensen, Elizabeth R. Gerstner, Gordon Harris, Ian E. Krop, Daniel Morganstern, Jessica Sohl, Jiani Hu, Elizabeth Kasparian, Eric P. Winer, Nancy U. Lin

**Affiliations:** 1 Dana-Farber Cancer Institute, Dana-Farber/Brigham & Women’s Cancer Center, 450 Brookline Avenue, Boston, MA 02215 USA; 2 Department of Diagnostic Physics, Oslo University Hospital, Oslo, Norway; 3 Indiana University School of Medicine, Indianapolis, IN USA; 4 Massachusetts General Hospital, Boston, MA USA; 5 University of Texas Southwestern Medical Center, Dallas, TX USA; 6 IMRIS Inc., Minnetonka, MN USA

**Keywords:** Breast cancer, Brain metastases, Bevacizumab

## Abstract

**Background:**

We aimed to examine the safety and efficacy of bevacizumab and carboplatin in patients with breast cancer brain metastases.

**Methods:**

We enrolled patients with breast cancer and > 1 measurable new or progressive brain metastasis. Patients received bevacizumab 15 mg/kg intravenously (IV) on cycle 1 day 1 and carboplatin IV AUC = 5 on cycle 1 day 8. Patients with HER2-positive disease also received trastuzumab. In subsequent cycles, all drugs were administered on day 1 of each cycle. Contrast-enhanced brain MRI was performed at baseline, 24–96 h after the first bevacizumab dose (day + 1), and every 2 cycles. The primary endpoint was objective response rate in the central nervous system (CNS ORR) by composite criteria. Associations between germline VEGF single nucleotide polymorphisms (rs699947, rs2019063, rs1570360, rs833061) and progression-free survival (PFS) and overall survival (OS) were explored, as were associations between early (day + 1) MRI changes and outcomes.

**Results:**

Thirty-eight patients were enrolled (29 HER2-positive, 9 HER2-negative); all were evaluable for response. The CNS ORR was 63% (95% CI, 46–78). Median PFS was 5.62 months and median OS was 14.10 months. As compared with an Eastern Cooperative Oncology Group performance status (ECOG PS) of 0, patients with ECOG PS 1–2 had significantly worse PFS and OS (all *P* < 0.01). No significant associations between VEGF genotypes or early MRI changes and clinical outcomes were observed.

**Conclusions:**

The combination of bevacizumab and carboplatin results in a high rate of durable objective response in patients with brain metastases from breast cancer. This regimen warrants further investigation.

**Trial registration:**

NCT01004172. Registered 28 October 2009.

## Introduction

Up to half of patients with human epidermal growth factor receptor 2 (HER2)-positive or triple-negative metastatic breast cancer, and 10–15% of patients with estrogen receptor (ER)-positive/HER2-negative metastatic breast cancer, will develop brain metastases [1, 2]. The management of patients with brain metastases is a continuing challenge.

Brain metastases from breast cancer are highly vascular and exhibit abnormal vessel morphology [3]. Preclinical data suggest that vascular endothelial growth factor (VEGF) promotes the growth of breast cancer brain metastases and that treatment with antiangiogenic agents results in tumor regression [4, 5]. Bevacizumab is a humanized, monoclonal antibody directed against VEGF. At the time the study was designed, improvements in the objective response rate (ORR) and progression-free survival (PFS) had been reported among patients with HER2-negative metastatic breast cancer when bevacizumab was added to chemotherapy, and dual antibody blockade with bevacizumab and trastuzumab was shown to produce objective responses in HER2-positive disease [6, 7]. Evidence from autopsy series indicates that platinum agents reach therapeutic concentrations in brain metastases [8]. The combination of cisplatin and etoposide has been reported in a small study to lead to a central nervous system (CNS) ORR of 38% in breast cancer patients, albeit in a patient population that was less refractory (no prior cranial radiotherapy, more than two thirds of patients chemotherapy-naive) than typical contemporary patients [9]. In addition, preclinical data suggest a possible synergism between bevacizumab and carboplatin in glioma models [10].

Since this study was initiated, multiple randomized studies have failed to demonstrate an overall survival benefit for bevacizumab in metastatic breast cancer and regulatory approval in the USA was withdrawn [11–15]. However, patients with active brain metastases were excluded due to concerns about intracranial hemorrhage. Thus, whether brain metastases may be particularly sensitive to angiogenic blockade remains an understudied question.

In this study, we evaluated the safety and efficacy of bevacizumab and carboplatin in breast cancer patients with new or progressive brain metastases. We also explored several potential predictors of outcome, including VEGF genotype and early imaging changes.

## Patients and methods

### Patients

We enrolled 38 patients with metastatic breast cancer and measurable CNS disease (> 1 parenchymal brain lesion with longest diameter > 10 mm), new or progressive CNS lesion(s), Eastern Cooperative Oncology Group performance status (ECOG PS) 0–2, no increase in corticosteroid dose in the week prior to the baseline brain MRI, left ventricular ejection fraction > 50%, and adequate organ function. Patients could have received prior carboplatin if not given with bevacizumab. Prior bevacizumab was allowed if it had not been given within 6 months prior to the diagnosis of CNS metastases. Prior whole brain radiation therapy (WBRT) and/or stereotactic radiosurgery (SRS) was allowed but not required. For patients who had received prior radiation therapy (RT), patients had to have developed subsequent CNS progression or the presence of residual untreated lesion(s) (which would be followed as target lesion[s]). There was no limit on the number of prior therapies. Key exclusion criteria were contraindication to MRI, leptomeningeal carcinomatosis as the only site of CNS involvement, and more than 2 seizures over the last 4 weeks prior to study entry. Bevacizumab-specific exclusions included uncontrolled hypertension, history of stroke or myocardial infarction within 6 months, New York Heart Association (NYHA) Class II or higher congestive heart failure, evidence of bleeding diathesis, need for full-dose anticoagulation, or major surgical procedure within 28 days.

### Study design

This was an open-label, nonrandomized, phase II study. Patients were enrolled into two simultaneously enrolling cohorts: HER2-negative and HER2-positive. In order to support the correlative MRI imaging endpoint, all patients received bevacizumab monotherapy 15 mg/kg intravenously on cycle 1 day 1 (C1D1). On C1D8, HER2-negative patients received carboplatin IV AUC = 5. HER2-positive patients also received trastuzumab (8 mg/kg load if indicated; otherwise, 6 mg/kg) on C1D8. Three weeks later, patients received both carboplatin (AUC = 5) and bevacizumab (15 mg/kg) on day 1 (plus trastuzumab 6 mg/kg, if HER2-positive) and every 3 weeks thereafter.

No dose modifications were allowed for bevacizumab or trastuzumab, which could be held in case of adverse events. For carboplatin, the protocol-specified dose holds and reductions were based upon both the absolute neutrophil count (ANC) and platelet count, as well as for grade 3/4 non-hematological toxicities. If one (or more) drug is needed to be discontinued, the patient could remain on protocol and the other drug(s) continued. Patients continued protocol therapy until disease progression in CNS and/or extracranial sites, unacceptable adverse events (AEs), physician’s judgment, or withdrawal of consent.

The protocol was approved by the Dana-Farber/Harvard Cancer Center (DF/HCC) Scientific Review Committee and Institutional Review Board. The study is registered at www.clinicaltrials.gov (NCT01004172). All study participants provided written informed consent prior to any study-related procedures.

### Study assessments

Patients were evaluated at baseline, C1D8, and day 1 of all subsequent cycles. A neurological examination worksheet was completed by the treating provider at each study visit. AEs were assessed according to the National Cancer Institute Common Terminology Criteria for Adverse Events version 3.0. Non-CNS restaging scans (CT or MRI) and brain MRI sequences were obtained at baseline and after every 2 cycles. If non-CNS disease remained stable after 4 cycles, the frequency of non-CNS imaging was decreased to once every 4 cycles. Left ventricular ejection fraction (LVEF) was assessed every 4 cycles.

The primary efficacy endpoint was CNS ORR, according to composite criteria. Protocol pre-specified response evaluation for this study was done using RECIST 1.0 for non-CNS lesions and composite criteria (which incorporated centrally assessed volumetric response) for CNS lesions according to previously published methods [16]. The MRI series used for response assessment of CNS lesions was T1-weighted gadolinium-enhanced. For imaging at all timepoints, MRI minimum requirements were to have 5 mm or less slice thickness for axial post-contrast images using T1 or SPGR sequence. Of note, all MRI scans at baseline, following the first dose of bevacizumab and prior to cycle 3, were performed centrally on the same machines at Massachusetts General Hospital Charlestown Navy Yard according to the following specifications, which included voxel size 0.5 × 0.5 × 0.8 (Supplemental Figure 1). All brain MRI scans were reviewed centrally by the DF/HCC Tumor Imaging Metrics Core. CNS partial response required *all* of the following: > 50% reduction in volumetric sum of all measurable brain metastases compared to baseline, no progression of non-measurable lesions, no new lesions, stable or decreasing steroid dose, no new/progressive tumor-related neurologic signs or symptoms, *and* no progression of non-CNS disease by RECIST 1.0. CNS complete response required the same items as partial response but with complete resolution of all measurable and non-measurable brain metastases. Patients were considered to have progressive disease if *any* of the following occurred: > 40% increase in the volumetric sum of all measurable lesions as compared to nadir, progression of non-measurable lesions, new lesion(s), increasing steroid requirement, new or progression tumor-related neurologic signs and symptoms, or progression of non-CNS disease by RECIST 1.0. Secondary clinical endpoints included safety/tolerability, PFS, clinical benefit rate (complete response + partial response + stable disease > 24 weeks), site of first progression, and overall survival (OS).

### Correlative studies

Blood for VEGF genotyping of four pre-specified VEGF single nucleotide polymorphisms (rs699947, rs2019063, rs1570360, rs833061) was collected at baseline. Associations between VEGF genotyping and PFS and OS were evaluated by log-rank tests.

MRI of the brain was performed on a 3 Tesla Siemens TrioTim, including conventional T2-weighted, fluid-attenuated inversion recovery (FLAIR) and T1-weighted imaging (with and without gadolinium). Imaging was performed at baseline, 24–96 h after C1D1 bevacizumab (day + 1), and after 2 cycles of treatment. Thirty-eight patients had evaluable MRIs. Contrast-enhancing lesions and areas of T2 abnormality on FLAIR images were outlined by an experienced neuroradiologist blinded to patient identity and time of visit by using a semi-automatic volumetric approach in *3D Slicer* [17]. The total number of image voxels formed the basis for the overall lesion volume.

### Statistical analysis

The HER2-positive and HER2-negative cohorts were analyzed together. All patients who received at least one dose of protocol therapy were included in the toxicity analysis. The analysis of objective response was limited to patients who received at least one dose of study treatment and who had measurable CNS disease, defined as at least one CNS lesion > 10 mm in longest dimension, confirmed by central radiology review. The study was designed to distinguish between a CNS response rate of 5% versus 20%, with responses in at least 1 of 12 assessable patients entered in the first stage in order to proceed to full accrual. At the end of the study, if responses were observed in > 4 of 37 assessable patients, the regimen would be declared worthy of further study. The study included a stopping rule for CNS hemorrhage. If 3 or more patients experienced a grade 2 or higher CNS bleeding event, the study would be terminated. With this design, there was a 73% chance of terminating the study if the true probability of CNS hemorrhage is 10%, and a 93% chance of early termination if the true probability is 15%.

Initially, the study was designed to accrue up to 20 HER2-positive and up to 20 HER2-negative patients, in order to explore the CNS response rate according to HER2 status. However, because of slow accrual to the HER2-negative cohort, the study was amended on August 23, 2011, to remove the limit on the number of maximum patients per cohort and to remove formal analyses of clinical outcomes by HER2 status.

PFS was defined as the time from start of treatment to time of progression, second cancer, or death, whichever occurred first. Patients who were alive and progression-free at the date of last contact were censored at the date of last contact. OS was defined as the time from start of treatment until death. Patients who were alive at the date of last contact were censored on that date. PFS and OS were estimated by Cox proportional hazards regression models with hazard ratios (HR) with 95% confidence intervals (CI) and presented by Kaplan-Meier plots. Significance level was *P* < 0.05 with Holm-Bonferroni corrections for multiple comparisons.

## Results

### Patients and treatment

Thirty-eight women were enrolled between November 2009 and August 2012 (29 HER2-positive, 9 HER2-negative). As shown in Table 1, 87% of patients were ECOG PS 0–1 on study entry, and patients had received a median of 3 lines of chemotherapy in the metastatic setting (range 0–10). Most patients (33/38; 87%) had previously received WBRT, SRS, or both, with median time from last radiation to study entry of 7.5 months. Among patients with HER2-positive disease, all but one had prior trastuzumab and 70% had prior lapatinib. Patients had to be on stable or decreasing doses of corticosteroids for at least 7 days prior to enrollment. Investigator-rated neurological signs and symptoms were collected throughout the study (Supplemental Figure 2). Half of patients were denoted as having at least one sign or symptom at study baseline. Seven patients (18.4%) were taking corticosteroids at baseline.

**Table 1:** Patient and tumor characteristics

Characteristic	All (***n*** = 38)	Cohort 1 (HER2-negative) (***n*** = 9)	Cohort 2 (HER2-positive) (***n*** = 29)
	No. of patients (%)	No. of patients (%)	No. of patients (%)
**Age, years**			
Median (range)	48 (31–62)	46 (31–57)	48 (32–62)
**Sex**			
Female	38 (100)	9 (100)	29 (100)
Male	0 (0)	0 (0)	0 (0)
**Race**			
White	34 (89)	8 (89)	26 (90)
Black or African-American	1 (3)	1 (11)	0 (0)
Asian	3 (8)	0 (0)	3 (10)
**Ethnicity**			
Hispanic or Latino	1 (3)	0 (0)	1 (3)
Non-Hispanic	37 (97)	9 (100)	28 (97)
**ECOG PS at baseline**			
0	24 (63)	4 (44)	20 (69)
1	9 (24)	3 (33)	6 (21)
2	5 (13)	2 (22)	3 (10)
**Stage at initial diagnosis**			
I	5 (13)	1 (11)	4 (14)
II	13 (34)	4 (44)	9 (31)
III	7 (18)	1 (11)	6 (21)
IV	13 (34)	3 (33)	10 (34)
**Receptor status at initial diagnosis**			
ER and/or PR+, HER2-negative	7 (18)	5 (56)	2 (7)^b^
ER and/or PR+, HER2-positive	14(37)	1 (11)^a^	13 (45)
ER and PR negative, HER2-positive	14(37)	0 (0)	14 (48)
ER, PR, and HER2-negative	3 (8)	3 (33)	0 (0)
**Disease-free interval**			
< 2 years	22 (58)	7 (78)	15 (52)
> 2 years	16 (42)	2 (22)	14 (48)
**Interval from metastatic diagnosis to CNS diagnosis** (median [months], range)	14.8 (0–107)	5.9 (0–44.7)	16.6 (0–107)
**Interval from CNS diagnosis to study entry** (median [months], range)	15.7 (0.2–63.6)	11.0 (0.4–25.5)	19.8 (0.2–63.6)
**Number of metastatic disease sites** (median, range)	4 (1–5)	4 (1–5)	4 (1–5)
**Sites of disease**			
CNS	38 (100)	9 (100)	29 (100)
Lung or pleural effusion	19 (50)	6 (67)	13 (45)
Liver	17 (45)	4 (44)	13 (45)
Bone	26 (68)	7 (78)	19 (66)
Breast or chest wall	5 (13)	1 (11)	4 (14)
Other	15 (39)	4 (44)	11 (38)
	Median (range)	Median (range)	Median (range)
**Baseline CNS disease volume (in cc)**	5.3 (0.7, 58.8)	8.2 (2.1, 24.7)	3.6 (0.7, 58.8)
	No. of patients (%)	No. of patients (%)	No. of patients (%)
**Number of target CNS lesions**			
1	9 (24)	2 (22)	7 (24)
2	14 (37)	2 (22)	12 (41)
3	7 (18)	3 (33)	4 (14)
4	6 (16)	1 (11)	5 (17)
5	2 (5)	1 (11)	1 (3)
	Median (range)	Median (range)	Median (range)
**Size of target CNS lesions (in mm)**	15.4 (10, 60.5)	16 (11.7, 40.9)	14.4 (10, 60.5)

^a^ One patient with ER-positive, HER2-positive breast cancer was entered in cohort 1 because of prior intolerance to trastuzumab and was treated with carboplatin and bevacizumab (without trastuzumab)

^b^Two patients had ER-positive, HER2-negative breast cancer at diagnosis but subsequent biopsy at recurrence showed HER2-positive disease

Abbreviations: *CNS* central nervous system, *ECOG PS* Eastern Cooperative Oncology Group Performance Status, *ER* estrogen receptor, *HER2* human epidermal growth factor receptor 2, *PR* progesterone receptor

At time of data-lock (June 1, 2017), no patients were still receiving protocol therapy. The reasons for protocol therapy discontinuation were progression in CNS only (*n* = 16), progression in non-CNS only (*n* = 16), progression in both CNS and non-CNS (*n* = 2), treatment-related toxicity (*n* = 1), physician or patient decision (*n* = 1), and symptomatic deterioration (*n* = 2).

### Efficacy

**Fig. 1:**
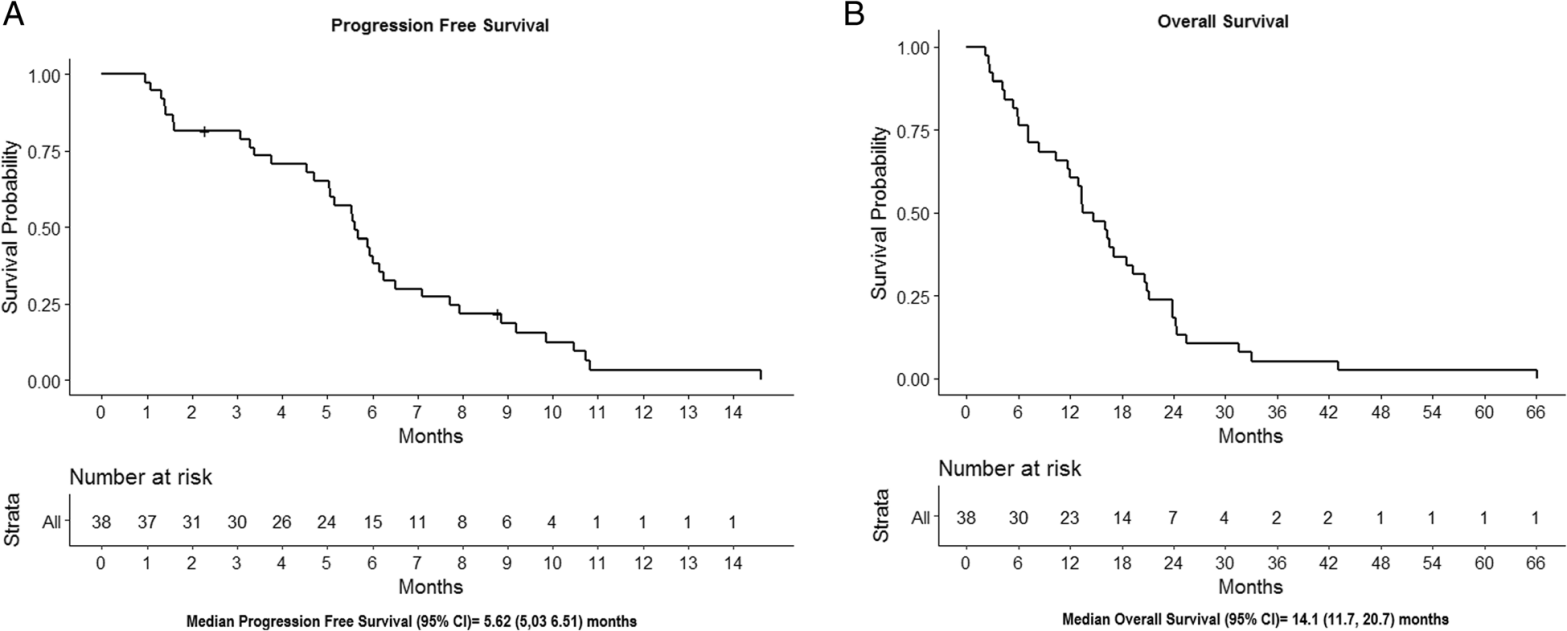
Progression-free survival (**a**) and overall survival (**b**) Kaplan-Meier plot. Two patients came off-treatment before disease progression. They were censored at the off-treatment time point

**Fig. 2:**
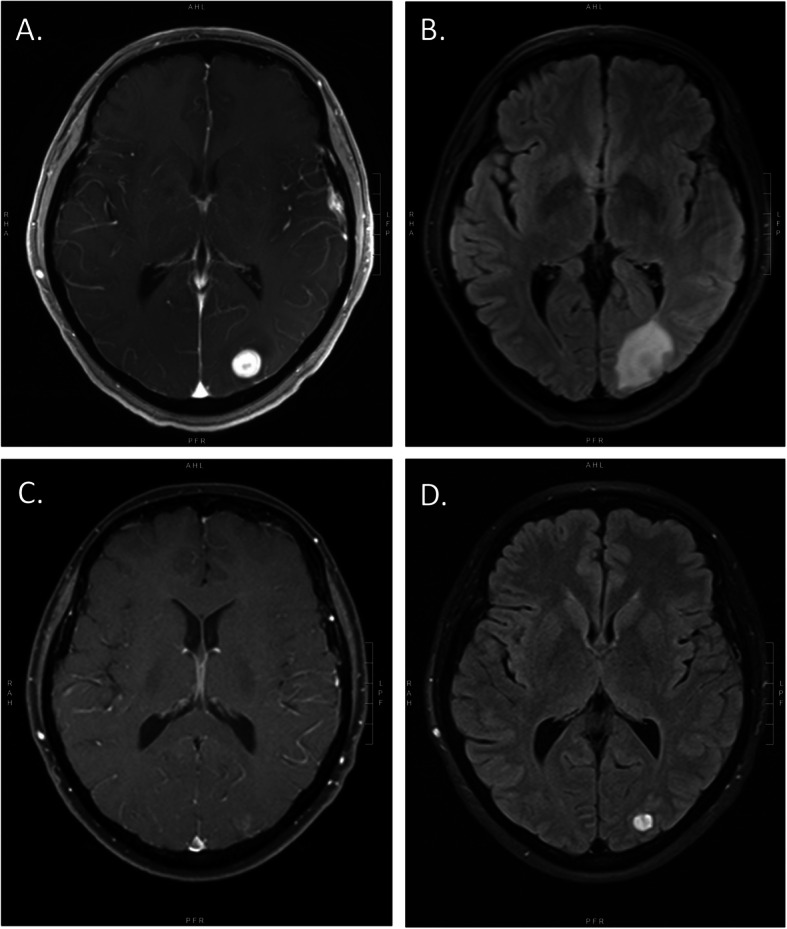
Brain MRI of a representative study participant at baseline, showing axial, T1-weighted, post-gadolinium (**a**) and T2-FLAIR (**b**) images. Brain MRI of the same participant after 10 months of protocol therapy, showing axial, T1-weighted, post-gadolinium (**c**) and T2-FLAIR (**d**) images, consistent with response to treatment

As shown in Table 2, according to protocol pre-specified composite criteria for response, the CNS ORR in the 38 assessable patients was 63% (95% CI, 47–77%). Responses were seen in both HER2-positive and HER2-negative patients. In a post hoc analysis, we also calculated the CNS ORR by response assessment in neuro-oncology brain metastases (RANO-BM) criteria and noted a CNS ORR of 47% [18]. Of the 27 patients with measurable non-CNS disease at baseline, objective non-CNS responses were observed in 31% of patients overall, but at a higher frequency in HER2-positive patients. Median PFS (which included both CNS and non-CNS progression events) was 5.62 months (95% CI, 5.03–6.51 months) (Fig. 1a). At the time of the data-lock, all patients had died. Median OS was 14.1 months (95% CI, 11.7–20.7 months) (Fig. 1b). Figure 2 shows the MRI of a patient at baseline and after 10 months of protocol therapy, showing partial response of her left occipital lesion from 2.99 cc (16.4 mm) at baseline to 0.59 cc (9.1 mm).

**Table 2:** Summary table of CNS best response by composite criteria among evaluable patients

Response	All (***n*** = 38)	Cohort 1 (HER2-negative) (***n*** = 9)	Cohort 2 (HER2-positive) (***n*** = 29)
	No. of patients (%)	No. of patients (%)	No. of patients (%)
CR	0 (0)	0 (0)	0 (0)
PR	24 (63)^a^	5 (56)	19 (66)
SD > 24 weeks	2 (5)	0 (0)	2 (7)
SD < 24 weeks	5 (13)	0 (0)	5 (17)
PD^b^	7 (18)	4 (44)	3 (10)
PD in CNS by radiographic criteria	1 (3)	0 (0)	1 (3)
Symptomatic deterioration	2 (5)	1 (11)	1 (3)
PD in non-CNS site(s)	4 (11)	3 (33)	1 (3)

^a^Taking into account the two-stage design, the 95% CI for CNS ORR (n = 38) is 47 to 77%

^b^Patients were considered to have progressed if any of the PD criteria were met. Each patient could also have been taken off study for meeting multiple PD criteria simultaneously

Abbreviations: *CNS* central nervous system, *CR* complete response, *HER2* human epidermal growth factor receptor 2, *PD* progression of disease, *PR* partial response, *SD* stable disease

### Safety

The most commonly reported AEs are listed in Table 3. Overall, the regimen was well tolerated. A total of 272 cycles of bevacizumab were administered, with a median of 8 cycles (range 1–20) per patient. The most common reason for dose delay or hold was hematological toxicity (thrombocytopenia, neutropenia without fever), which was most likely due to the carboplatin. Three cases of grade 1 CNS hemorrhage and one case of grade 2 CNS hemorrhage were reported (Supplemental Table 1). There were no cases of grade 3 or 4 CNS hemorrhage. Carboplatin dose reductions were required in 23 patients. The most common reason for carboplatin dose reduction was thrombocytopenia (*n* = 22). Cardiac function was assessed every 4 cycles on study. Asymptomatic declines in LVEF < 50% were observed in 5 (13%) patients, with nadir ranging from 44 to 48%.

**Table 3:** Summary of adverse events related to treatment with at least 10% incidence—by cohort

	Overall	Cohort						
		HER2-negative (*N* = 9)			HER2-positive (*N* = 29)			
		Grade			Grade			
	N	1	2	3	1	2	3	4
**AE name**								
*Hemoglobin*	29 (76%)	4 (44%)	3(33%)		10 (34%)	11 (38%)	1 (3%)	
*Fatigue*	28 (74%)	1 (11%)	3(33%)	2(22%)	9 (31%)	9 (31%)	4 (14%)	
*Platelets*	28 (74%)	3 (33%)	3(33%)		6 (21%)	12 (41%)	3 (10%)	1 (3%)
*AST*	23 (61%)	5 (56%)	1(11%)		15 (52%)		2 (7%)	
	22 (58%)	2 (22%)	1(11%)		3 (10%)	13 (45%)	3 (10%)	
*Head/headache*	20 (53%)	4 (44%)			10 (34%)	6 (21%)		
*Hyperglycemia*	18 (47%)	3 (33%)	1(11%)		12 (41%)	2 (7%)		
*Leukocytes*	18 (47%)				4 (14%)	14		
*Nausea*	17 (45%)	1 (11%)	1(11%)		13 (45%)	2 (7%)		
*ALT*	14 (37%)	3 (33%)	1(11%)		8 (28%)		2 (7%)	
*Constipation*	14 (37%)	3(33%)	1(11%)		7 (24%)	3 (10%)		
*Neuropathy-sensory*	14 (37%)	3(33%)			9 (31%)	2 (7%)		
*Nose, hemorrhage*	12 (32%)	1(11%)			9 (31%)	2 (7%)		
*Alkaline phosphatase*	11 (29%)				9 (31%)	2 (7%)		
*Diarrhea w/o prior colostomy*	11 (29%)	1(11%)	1(11%)		7 (24%)	2 (7%)		
*Cough*	9 (24%)				8 (28%)	1 (3%)		
*Hypertension*	9 (24%)				6 (21%)	2 (7%)	1 (3%)	
*Alteration in bone age*	8 (21%)				6 (21%)	2 (7%)		
*Proteinuria*	8 (21%)	1(11%)			6 (21%)	1 (3%)		
*Extremity-limb, pain*	7 (18%)				4 (14%)	2 (7%)	1 (3%)	
*Alopecia*	6 (16%)		1(11%)			4 (14%)	1 (3%)	
*Hyponatremia*	6 (16%)				5 (17%)		1 (3%)	
*Joint, pain*	6 (16%)				3 (10%)	2 (7%)	1 (3%)	
*Vomiting*	6 (16%)	2**(22%)**	1(11%)		2 (7%)	1 (3%)		
*Allergic reaction*	5 (13%)				1 (3%)	4 (14%)		
*Back, pain*	5 (13%)				4 (14%)	1 (3%)		
*Dizziness*	5 (13%)				2 (7%)	3 (10%)		
*Infection Grade 0–2, upper airway*	5 (13%)				4 (14%)	1 (3%)		
*Left ventricular systolic dysfunction*	5 (13%)					5 (17%)		
*Pain-other*	5 (13%)	1(11%)	1(11%)		1 (3%)	2 (7%)		
*Rectum, hemorrhage*	5 (13%)				4 (14%)	1 (3%)		
*Bone, pain*	4 (11%)				4 (14%)			
*CNS, hemorrhage*	4 (11%)	1(11%)	1(11%)		2 (7%)			
*Constitutional, other*	4 (11%)				3 (10%)	1 (3%)		
*Creatinine*	4 (11%)	2(22%)			2 (7%)			
*GI-other*	4 (11%)				4 (14%)			
*Hypocalcemia*	4 (11%)				4 (14%)			
*Hypokalemia*	4 (11%)	1(11%)			3 (10%)			
*Metabolic/laboratory-other*	4 (11%)	2(22%)	1(11%)		1 (3%)			
*Skin-other*	4 (11%)	2(22%)			1 (3%)	1 (3%)		
*Throat/pharynx/larynx, pain*	4 (11%)				4 (14%)			

Abbreviations: *AE* adverse event, *ALT* alanine aminotransferase, *AST* aspartate aminotransferase, *CNS* central nervous system, *GI* gastrointestinal, *HER2* human epidermal growth factor receptor 2

### VEGF genotyping

DNA was extracted from 29 blood samples. No significant association was found between VEGF genotypes and PFS or OS (Supplemental Table 2).

### Early MRI findings

A brain MRI was performed between 24 and 96 h after C1D1 of bevacizumab (day + 1). The goal of this analysis was to assess whether changes from baseline volume to volume at day + 1 would be associated with PFS and OS. To explore this association, we divided the patient cohort by the median into two groups that were compared: a group who had below the median decrease in volume of contrast-enhancing lesions on day + 1, and a group who had above the median decrease in volume of contrast-enhancing lesions on day + 1.

**Fig. 3:**
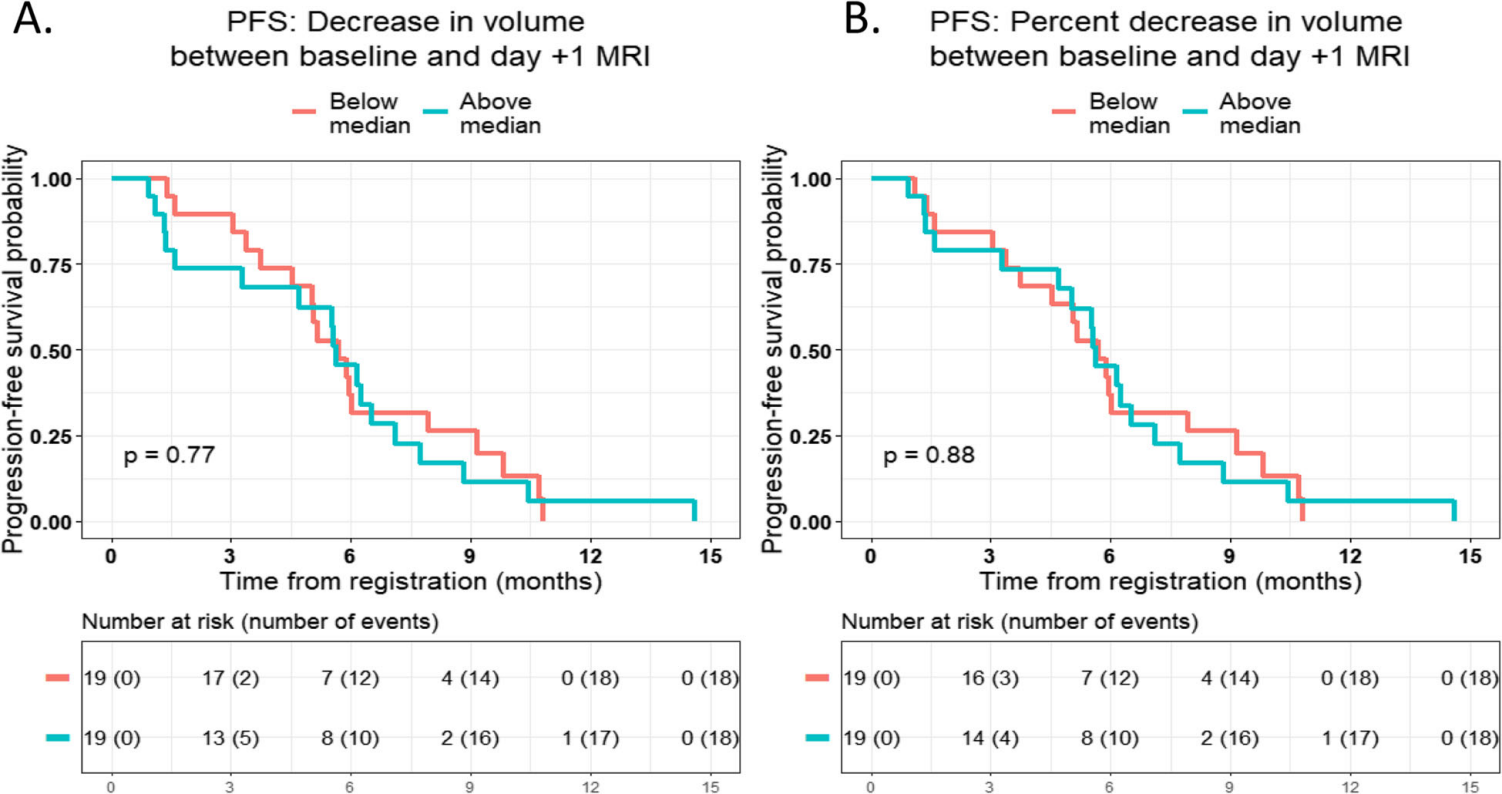
Association between progression-free survival (PFS) and **a** absolute decrease in volume between baseline MRI and day + 1 MRI, and **b** relative decrease in volume between baseline MRI and day + 1 MRI

Figure 3 shows the Kaplan-Meier curves for PFS by the degree of decrease in volume between baseline MRI and day + 1 MRI. There were no significant differences in PFS between patients who had below the median decrease in volume and those who had above the median decrease in volume, both when absolute decrease in volume was measured (*P* = 0.77; Fig. 3a) as well as when relative decrease in volume was measured (*P* = 0.88; Fig. 3b).

**Fig. 4:**
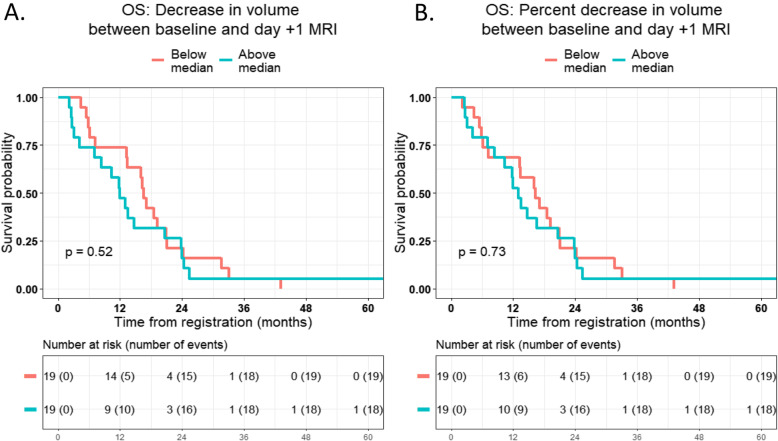
Association between overall survival (OS) and **a** absolute decrease in volume between baseline MRI and day + 1 MRI, and **b** relative decrease in volume between baseline MRI and day + 1 MRI

We observed similar findings for OS (Fig. 4). Patients who had below the median decrease in volume had no significant differences in OS compared with those who had above the median decrease in volume, both in terms of absolute decrease in volume (*P* = 0.52; Fig. 4a) as well as relative decrease in volume (*P* = 0.73; Fig. 4b).

We subsequently conducted landmark analyses for PFS and OS at the first follow-up MRI and at the second follow-up MRI. There were no significant associations between the degree of volume decrease by the first follow-up MRI and PFS or OS, nor between the degree of volume decrease by the second follow-up MRI and PFS or OS (data not shown).

The only variable with significant associations with PFS and OS was ECOG PS at baseline. As compared with ECOG PS = 0, patients with ECOG PS = 1 (HR = 4.65; *P* = 0.001) and ECOG PS = 2 (HR = 9.71; *P* < 0.001) had significantly worse PFS. Similarly, as compared with ECOG PS = 0, patients with ECOG PS = 1 (HR = 5.37; *P* = 0.001) and ECOG PS = 2 (HR = 18.67; *P* < 0.001) experienced significantly worse OS.

## Discussion

We evaluated the safety and efficacy of carboplatin and bevacizumab in patients with new or progressive breast cancer brain metastases. We found that the regimen was generally well tolerated and associated with a CNS ORR of 63% (95% CI 47–77) by pre-specified volumetric criteria, with responses seen across breast cancer subtypes. CNS ORR calculated retrospectively using RANO-BM criteria was 47%.

Since our study was initiated, the role of bevacizumab in metastatic breast cancer has undergone a major evolution, and its U.S. regulatory approval was revoked in November 2011 [11, 13, 14, 19–21]. Despite this, it is important to note that none of the phase III trials included patients with active brain metastases. We believe our results support further evaluation of bevacizumab in the treatment of patients with breast cancer brain metastases. Our efficacy results are consistent with data from a phase II trial conducted in Taiwan evaluating bevacizumab, etoposide, and cisplatin in breast cancer patients with progressive CNS after WBRT [22]. Our results are also in line with a phase II trial of patients with brain metastases from non-small cell lung cancer, where the combination of bevacizumab with carboplatin and paclitaxel achieved a CNS ORR of 61.2% [23]. Historical data with the combination of lapatinib plus capecitabine using identical response criteria have indicated a CNS response rate of 18–38% in patients with HER2-positive breast refractory cancer [16, 24–27]. The CNS response rate to lapatinib plus capecitabine was higher (67%) in the upfront setting, but in a less heavily pre-treated population with no prior radiotherapy [28]. In our study, 87% of patients had progressed after radiotherapy, and 70% of HER2-positive patients had prior lapatinib. Furthermore, the CNS ORR we observed also compares favorably with that of neratinib and capecitabine (CNS ORR 49%) and tucatinib-trastuzumab-capecitabine (CNS ORR 47%) [29, 30]. For patients with HER2-negative disease, options are substantially more limited. In TBCRC 018, the CNS ORR in patients with TNBC was 12% [31]. In the JPBO study, the CNS ORR in patient patients with ER+/HER2-negative disease was 5.8% [32]. In addition, we observed a median OS of 14.10 months in the study overall, which compares favorably to historical data, where median survival in patients after progression through WBRT is 6 months or less [16, 33–35]. However, cross-trial comparisons must be interpreted with caution.

The rate of CNS hemorrhage seen in our study is consistent with the rates observed in prior reports [36]. These suggest that the use of bevacizumab-containing regimens is safe in patients with brain metastases from breast cancer. A similar low rate of CNS hemorrhage was seen with the use of bevacizumab in patients with brain metastases from non-small cell lung cancer [37].

Because bevacizumab can rapidly normalize vascular permeability and thus reduce gadolinium leakiness, one potential critique is that the high CNS response rate we observed in our study could be related to purely radiological changes that do not necessarily confer clinical benefit [38, 39]. However, only patients who also had simultaneous non-CNS disease stability or response were counted as CNS responders per our pre-specified definitions, supporting that patients were deriving clinical benefit. Finally, we did observe extracranial responses in a proportion of patients who had measurable extracranial metastases at study entry. While we did not identify a significant association between decrease in volume of CNS lesions and PFS or OS, these results should be interpreted with caution, as the number of patients for subgroup comparisons was small.

Our study had several limitations. First, we did not include bevacizumab-alone or carboplatin-alone arms and therefore cannot determine the individual contributions of these agents, although our efficacy results did exceed those of historical data with platinum alone [6, 7, 9, 40, 41]. Second, we cannot exclude the possibility that trastuzumab may have contributed to the CNS response rate in patients with HER2-positive disease. Although we did observe responses in all major breast cancer subtypes, we are not able to formally compare outcomes by subtype. In addition, we did not include neurocognitive testing or patient-reported outcomes, which would have been a valuable addition in light of uncertainty regarding the meaning of radiographic responses in the setting of antiangiogenic therapies [42]. Similarly, presence or absence of edema was not quantified as part of this trial. However, the pre-specified response criteria used in the study provided a structured format for response evaluation. Finally, although we were not able to identify associations between VEGF genotypes and clinical outcomes, we acknowledge that our sample size was small, and our power limited.

## Conclusion

We report a high rate of durable, objective CNS responses with the combination of bevacizumab with carboplatin in patients with breast cancer brain metastases, and that MRI changes detected as early as day + 1 after a single dose of bevacizumab were associated with long-term clinical outcomes, including overall survival. Given the dearth of available treatment options for such patients, we believe that further study of bevacizumab-based regimens is warranted, possibly in combination with novel agents targeting key pathways in brain metastases.

## Supplementary information

**Additional file 1.** Supplementary material containing supplemental tables and figures.

## Data Availability

The datasets used and/or analyzed during the current study are available from the corresponding author on reasonable request.

## Abbreviations

AE: Adverse event
ANC: Absolute neutrophil count
CI: Confidence interval
CNS: Central nervous system
ECOG PS: Eastern Cooperative Oncology Group performance status
ER: Estrogen receptor
HER2: Human epidermal growth factor receptor 2
HR: Hazard ratio
LVEF: Left ventricular ejection fraction
ORR: Overall response rate
OS: Overall survival
PFS: Progression-free survival
RANO-BM: Response assessment in neuro-oncology brain metastases
RT: Radiation therapy
SRS: Stereotactic radiosurgery
VEGF: Vascular endothelial growth factor
WBRT: Whole breast radiation therapy
