# Supplementary material for: Phase II trial of carboplatin and bevacizumab in patients with breast cancer brain metastases
Source: Breast Cancer Res. 2020 Nov 30;22:131. doi: 10.1186/s13058-020-01372-w (PMC7706261; doi:10.1186/s13058-020-01372-w)
Supplement: Supplementary file 1 — Additional file 1. Supplementary material containing supplemental tables and figures. [file 13058_2020_1372_MOESM1_ESM.pdf]

Supplemental Table 1. Details of CNS Hemorrhage Events Observed on Protocol

Patient	Summary of Event
Pt 025	Grade 1 asymptomatic punctate hemorrhage observed on restaging brain MRI prior to cycle 5 of therapy. Bevacizumab held; brain MRI repeated without progression of CNS bleeding, and bevacizumab resumed without incident.
Pt 034	Grade 1 petechial hemorrhage observed on early restaging brain MRI in setting of headaches. Was found to have evidence of tumor progression in liver, lungs, and brain. Transitioned to hospice shortly thereafter.
Pt 036	Grade 1 asymptomatic hemorrhage observed on restaging brain MRI prior to cycle 3 of therapy. Bevacizumab held; brain MRI repeated without progression of CNS bleeding, and bevacizumab resumed without incident.
Pt 027	Grade 2 hemorrhage presenting with altered mental status and paranoia. Bevacizumab permanently discontinued. Brain MRI ~2 months later demonstrated progressive CNS disease without worsening of CNS hemorrhage.

Supplemental Table 2. Relationship Between VEGF Single Nucleotide Polymorphisms and Clinical Outcomes. No significant association was found between genotypes with PFS or OS. The table shows the p values of the tests.

Clinical Outcome	-634 G/C (rs2010963)	-1498 C/T (rs833061)	-2578 C/A (rs699947)	-1154 G/A (rs1570360)
OS	0.54	0.56	0.76	0.54
PFS	0.75	0.39	0.34	0.75

Abbreviations: OS, overall survival; PFS, progression-free survival

Supplemental Figure 1. MRI/MRA Specifications

MRI:

MRI brain scans will be done at baseline, and at each follow-up. Brain MRI should have 5 mm or less slice thickness for axial post-contrast images using T1 or SPGR sequence.

MRI brain scans at baseline, following the first dose of bevacizumab, and prior to cycle 3 of treatment will be performed at MGH Charlestown Navy Yard. All other MRI brain scans may be performed at the patient's study site, according to the specifications in paragraph 1, above.

MRI brain scans at baseline, following bevacizumab, and prior to cycle 3 of treatment will include the following:

- T1- weighted images
- T2-weighted images
- FLAIR (fluid- attenuated inversion recovery)
- Arterial Spin Labeling (ASL) perfusion
- Contrast agent enhanced T1-weighted permeability
- DTI (diffusion tensor imaging)
- T2/T2*-weighted perfusion
- Modified MRA (See below)

Modified MRA:

- The entire brain should be covered. (The neck does not need to be covered.)
- The sequence should be obtained **PRIOR** to the administration of intravenous contrast.
- The following are suggested parameters

Seq	TR Msec	TE msec	Flip Angle	Matrix	FOV	Slice thick mm	Voxel Size mm3
MRA	35	3.5	22	309*448	230*230	0.8	0.5*0.5*0.8

Supplemental Figure 2. Neurological Examination Worksheet

NEUROLOGICAL EXAMINATION WORKSHEET

(to be completed at baseline and at the end of each 3-week cycle)

(pg. 1 of 2)

PLEASE NOTE IF SIGNS/SYMPTOMS ARE THOUGHT RELATED OR NOT RELATED TO PATIENT's BRAIN METASTASES

Patient________________________ Examiner_________________________Date____________

Level of consciousness (check one)

___Normal
___Somnolence or sedation not interfering with function
___Somnolence or sedation interfering with function, but not interfering with ADLs
___Obtundation or stupor; difficult to arouse; interfering with ADLs
___Coma

Neurological Symptoms (check if present, and specify CTCAE grade)

*if asymptomatic, check here _____

Headache	___present, specify grade____	
Dizziness/lightheadedness	___present, specify grade____	
Vertigo	___present, specify grade____	
Nausea/vomiting	___present, specify grade____	
Visual problems	___present, specify grade____	
Seizure	___present, specify grade____	
Other	___absent	___present, specify_____________grade_____

Cranial nerves II-XII+

___ Normal
___present, not interfering w/ADLs
___present, interfering w/ADLs
___life-threatening, disabling
+If abnormal, please specify which cranial nerve(s) affected___________________________________

Language

Dysphasia or aphasia ___absent
___awareness of receptive or expressive aphasia, not impairing ability to communicate
___receptive or expressive dysphasia, impairing ability to communicate
___inability to communicate

Sensation**

___normal
___loss of deep tendon reflexes or paresthesia, but not interfering with function
___objective sensory loss or paresthesia interfering with function, but not with ADLs
___Sensory loss or paresthesia interfering with ADLs
___Permanent sensory loss that interferes with function
***If abnormal, please specify location/distribution*____

NEUROLOGICAL EXAMINATION WORKSHEET
(pg. 2 of 2)

Patient________________________ Examiner_____________________ Date____________

Strength*

R upper extremity	__Normal	___Abnormal; please specify_______________
L upper extremity	__Normal	___Abnormal; please specify_______________
R lower extremity	__Normal	___Abnormal; please specify_______________
L lower extremity	__Normal	___Abnormal; please specify_______________

**If abnormal, please specify muscle group and grade according to the scale below: (e.g. biceps, grade 2)*

grade 1 = asymptomatic with weakness on physical exam
grade 2 = symptomatic and interfering w/function, but not interfering with ADLs
grade 3 = symptomatic and interfering with activities of daily living
grade 4 = bedridden or disabling

Ataxia***

R upper extremity (finger-to-nose testing)	___Normal	___Abnormal, specify grade ___
L upper extremity (finger-to-nose testing)	___Normal	___Abnormal, specify grade ___
Gait	___Normal	___Abnormal, specify grade ___
Balance (Romberg)	___Normal	___Abnormal, specify grade ___

***If any of above abnormal, please assign grade using the following criteria

grade 1 = asymptomatic but abnormal on physical exam, and not interfering with function
grade 2 = mild symptoms interfering with function, but not interfering with ADLs
grade 3 = moderate symptoms interfering with ADLs
grade 4 =bedridden or disabling

In the opinion of the treating physician, overall, are the patient's *tumor-related* neurological signs and symptoms worsening, stable, or improved (please check one)?

___ Worsening
___ Stable
___ Improved

Is the patient currently taking corticosteroids?

___Yes
___ No

If yes, please list name of medication and dose (e.g. decadron, 4 mg QD)______________

Please indicate the patients ECOG Performance Status: ____
